# Decoding the Spitz Puzzle: Histological Patterns and Diagnostic Challenges in Everyday Pathology Practice—A Single-Center Study

**DOI:** 10.3390/medicina61081501

**Published:** 2025-08-21

**Authors:** Iuliu Gabriel Cocuz, Georgian-Nicolae Radu, Maria Cătălina Popelea, Raluca Niculescu, Maria Elena Cocuz, Adrian-Horațiu Sabău, Andreea-Cătălina Tinca, Andreea Raluca Cozac-Szoke, Bogdan Pastor, Diana Maria Chiorean, Corina Eugenia Budin, Irina Bianca Kosovski, Ovidiu Simion Cotoi

**Affiliations:** 1 Pathophysiology Department, “George Emil Palade” University of Medicine, Pharmacy, Sciences and Technology of Targu Mures, 540142 Targu Mures, Romania; iuliu.cocuz@umfst.ro (I.G.C.); raluca.niculescu@umfst.ro (R.N.); adrian-horatiu.sabau@umfst.ro (A.-H.S.); andreea-catalina.tinca@umfst.ro (A.-C.T.); andreea-raluca.szoke@umfst.ro (A.R.C.-S.); chioreandianamaria@yahoo.com (D.M.C.); corina.budin@umfst.ro (C.E.B.); bianca.kosovski@umfst.ro (I.B.K.); ovidiu.cotoi@umfst.ro (O.S.C.); 2 Clinical Pathology Department, Mures Clinical County Hospital, 540011 Targu Mures, Romania; popelea.maria@gmail.com (M.C.P.); bogdipastor@yahoo.com (B.P.); 3 Histology Department, “George Emil Palade” University of Medicine, Pharmacy, Sciences and Technology of Targu Mures, 540142 Targu Mures, Romania; 4 Fundamental Prophylactic and Clinical Disciplines Department, Faculty of Medicine, Transilvania University of Brasov, 500003 Brașov, Romania; maria.cocuz@unitbv.ro; 5 Clinical Pneumology and Infectious Diseases Hospital of Brasov, 500174 Brasov, Romania; 6 Pneumology Department, Mures Clinical County Hospital, 540011 Targu Mures, Romania; 7 Clinical Laboratory Department, Mures Clinical County Hospital, 540011 Targu Mures, Romania

**Keywords:** Spitz tumors, histopathology, immunohistochemistry, diagnostic challenges

## Abstract

*Background and Objectives*: Spitz tumors represent a diagnostic challenge in dermatopathology due to their large spectrum of morphological characteristics and overlap with malignant lesions, especially in pathology departments where molecular pathology is not available. Even though most Spitz lesions are benign, the uncertainty around their biological behavior necessitates an integrated approach in daily practice. The objective of our study was to evaluate the epidemiological, macroscopic, and histopathological characteristics of Spitz lesions in accordance with *WHO Classification of Skin Tumours*. *Materials and Methods*: We performed a retrospective, descriptive, and hypothesis-generating study on Spitz tumors diagnosed between 2018 and 2024 in the Clinical Pathology Department of the Mures Clinical County Hospital, Romania. We included 10 cases and analyzed their macroscopic characteristics (localization, shape, dimension, and color), microscopic characteristics (cellular types, cytologic atypia, pagetoid migration, mitoses, and the type of lesion), and immunohistochemical profile. *Results*: The study population was composed of young patients with an average age of 20.2 years old, with a slight predominance of female gender. Most lesions were Spitz nevi, intradermic, or compound, with a fusiform, epithelioid, or rhomboid cell shape. Pagetoid migration and cytological atypia were seen in fewer cases. The Ki 67 proliferation index was under 5% in all cases. The main limitation of this study involved the low number of cases and the lack of molecular testing, which limited the molecular characterization of Spitz tumors. Complete excision was performed in all cases. *Conclusions*: In the absence of molecular testing, our study emphasizes the importance of clinical–morphological assessment using immunohistochemistry in establishing a correct diagnosis in Spitz lesions. Our results confirm that most of the Spitz lesions were benign and provide a basis for future research with a multidisciplinary approach, including molecular testing.

## 1. Introduction

Melanocytic nevi represent one of the most common benign skin tumoral proliferations in humans [1]. Melanocytic tumors are described as benign, malignant, or borderline lesions, mostly depending on their histological appearance. Nevi, as melanocytic skin tumors, mostly belong in the benign category [1]. The Spitz nevus was first described by Sophie Spitz as the “benign nevus of childhood” in 1947 and further developed into the category of Spitz tumors. More than 70 years after their first documentation, Spitz tumors remain rare and are diagnosed mostly in children and young patients [2,3].

Spitz tumors are represented by benign tumoral proliferations (Spitz nevi), borderline tumoral proliferations with an uncertain malignant potential (Spitz melanocytoma or atypical Spitz tumor), and malignant tumoral proliferations (Spitz melanoma) [1,4]. Depending on their clinical features and histopathological, immunohistochemical, and molecular profiles, these three categories of Spitz tumors vary in terms of prognosis and rate of recurrence.

Spitz nevi are represented by many different variants, as described in the literature, with various histological patterns, but they maintain a benign appearance, in contrast to Spitz melanocytoma, which usually has an indolent character, or Spitz melanoma, which represents the malignant category of Spitz tumors, with a lower rate of occurrence and a poor prognosis.

Depending on patient characteristics, the prognosis and clinical course of Spitz tumors can vary significantly. A histopathological examination, supported by immunohistochemistry, remains fundamental. However, emerging evidence has shown that molecular profiling is crucial in accurate diagnosis and prognosis stratification. Recent molecular studies have demonstrated that up to 80–90% of Spitz tumors harbor gene fusions involving ALK, ROS1, NTRK1/2/3, BRAF, RET, or MAP3K8, activating the MAPK or PI3K-AKT pathways [5,6,7]. These genetic alterations not only support histopathological subtyping but also help distinguish Spitz nevi from atypical Spitz tumors and Spitz melanomas. Notably, certain mutations (e.g., TERT promoter mutations, CDKN2A deletion, or KMT2C mutations) have been associated with progression or malignancy [8,9]. In our study, although limited by the lack of molecular testing, we underscore the diagnostic value of integrating morphological and immunohistochemical features, offering practical guidance for resource-limited pathology settings. This adds to the current literature by characterizing Spitz lesions in a real-world, low-resource diagnostic context.

In terms of molecular classification, approximately 80% of Spitz lesions have gene fusions (ALK, ROS1, NTRK1/2/3, BRAF, etc.), leading to the consecutive activation of the MAPK pathway. Advanced or atypical Spitz tumors may present TERT promoter mutations, associated with metastatic progression or epigenetic mutations (KMT), suggesting a possible progressive genomic instability [5,10,11].

Spitz tumors pose ongoing diagnostic challenges, particularly in settings without molecular testing capabilities, making the integration of clinical and histological data essential. This way, the characteristics of each patient and lesion serve as the main reference for a correct diagnosis. Table 1 presents the main differences between Spitz lesions.

## 2. Materials and Methods

A retrospective study was performed based on the histopathological diagnosis of Spitz nevi or atypical Spitz tumors (ASTs). The study included 10 cases of Spitz tumors that were diagnosed in the Clinical Pathology Department of the Mures Clinical County Hospital, Romania, between 2018 and 2024, but no cases were diagnosed in 2024. The inclusion criterion was a histopathological diagnosis of any Spitz tumor in our pathology department. The exclusion criteria were based on any other diagnosis that had not met the inclusion criteria. All cases from our pathology department involving Spitz tumors were included.

The tissue samples were received by the pathology department from either the General Surgery ward or the Plastic Surgery ward from patients diagnosed with a skin tumoral proliferation who had undergone the surgical excision of the lesions. The tissue samples were fixed in 10% histological formaldehyde and were then processed according to the routine protocol for histopathological processing. Hematoxylin and Eosin staining was performed based on the routine protocol using Epredia Gemini™ Stainer (Epredia, Kalamazoo, MI, USA, 2018). The immunohistochemistry assessment was performed using the Roche Ventana BenchMark (Ventana Medical Systems, Inc., now Roche Tissue Diagnostics, Tucson, AZ, USA, 2021) automated staining system.

The slides were examined by 2 consultant pathologists with experience in dermatopathology, and a histopathological diagnosis of one of the Spitz tumors was established.

The data were collected from the histopathological reports prepared after microscopic examination. The epidemiological data were represented by the year of diagnosis, age of the patient, and gender. The macroscopic data were represented by the localization of the lesion, macroscopic appearance, macroscopic dimension (mm)/diameter or circumference (mm^3^/mm), macroscopic shape, and macroscopic color. The microscopic parameters analyzed included the cell shape, the presence or absence of cytological atypia, pagetoid migration, the presence or absence of mitoses, the presence or absence of junctional components, and the immunohistochemistry performed. Macroscopic and microscopic lesions were also assessed, together with the final histopathological diagnostics. The data were represented and analyzed using Microsoft Office suite. Statistical analysis was performed using GraphPad Prism 10.5.0 (GraphPad Software, Inc., San Diego, CA, USA). The Pearson correlation coefficient was used for the evaluation of the relationships between the studied variables in the case of numeric or binary variables. Every correlation was represented by the r coefficient together with the *p* value (two-tailed) for statistical significance. Due to the small number of cases included in the study, the results should be interpreted with caution. The small cohort represents a limitation in the statistical value of extrapolating the results to a larger scale. Moreover, type I errors (a false-positive effect) or type II errors (the impossibility of the detection of a real effect) can appear. This study is predominantly descriptive.

Photography, image editing, and the addition of the scale to each picture was performed with Zeiss Axio Lab 1 (Carl Zeiss Microscopy GmbH, Jena, Germany, 2017) and ZEN 3.2 (blue edition) (Carl Zeiss Microscopy GmbH, Jena, Germany, 2023).

This study was conducted in accordance with the Declaration of Helsinki and approved by the Ethics Committee of the Mures Clinical County Hospital (protocol code 12744/9 September 2024).

## 3. Results

This study included 10 cases of tumors that had the histopathological diagnosis of a Spitz tumor.

**Figure 1:**
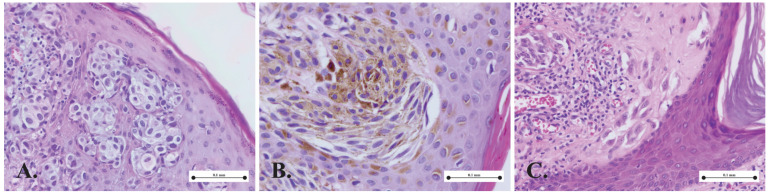
Cellular morphology in Spitz nevus: (**A**) nests of epithelioid melanocytes; (**B**) junctional nest composed of spindle melanocytes; (**C**) cluster of rhomboid melanocytes.

**Figure 2:**
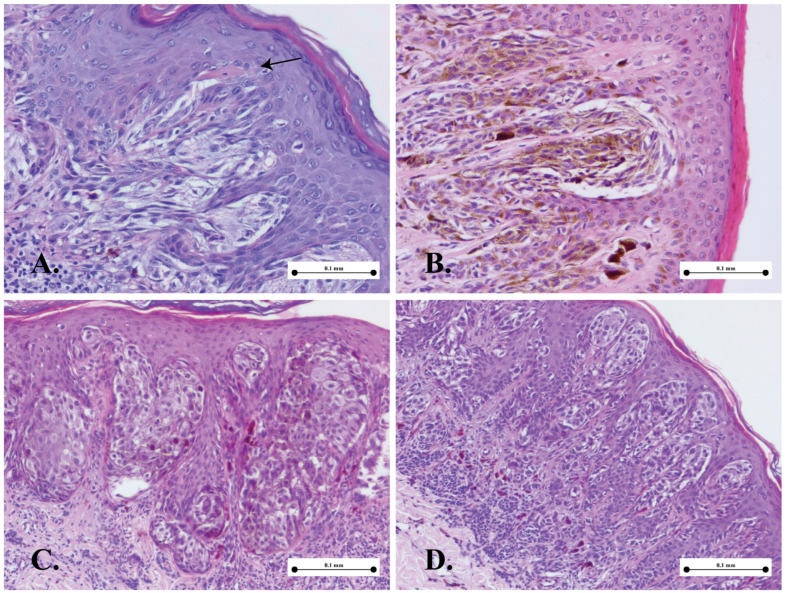
Histological features in Spitz nevus. (**A**) Kamino bodies—eosinophilic globules, usually found in the dermo-epidermal junction (arrow); (**B**) vertically oriented junctional nest with clefting artifact; (**C**) pagetoid spread; (**D**) maturation.

**Figure 3:**
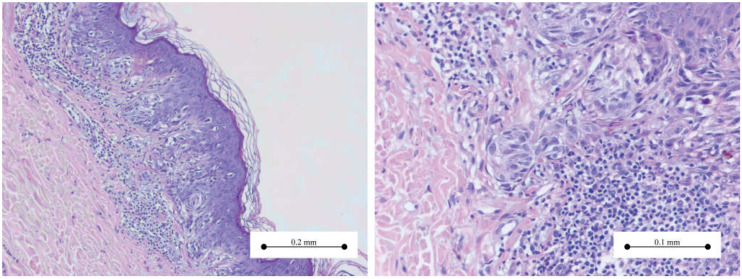
(**Left**) Compound Spitz nevus—symmetric melanocytic proliferation confined to the dermo-epidermal junction and underlying papillary dermis. (**Right**) Intradermal component—spindle-shaped melanocytes, arranged in small nests in the superficial dermis.

**Figure 4:**
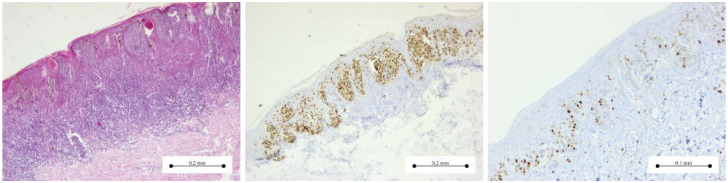
Compound Spitz nevus: (**Left**) Hematoxylin and Eosin staining—Melanocytic proliferation of spindle-shaped cells confined to the dermo-epidermal junction and superficial layer of dermis; (**Middle**) SOX-10 immunohistochemistry marker—intensely, diffused nuclear staining pattern in the spindle melanocytes; (**Right**) Ki-67 immunohistochemistry—positive staining in few junctional and intradermal melanocytes—Ki-67 index of 1–2% in the melanocytic proliferation.

Figure 1, Figure 2, Figure 3 and Figure 4 present the morphological and immunohistochemical characteristics of Spitz tumors. All images are from cases included in this study.

**Figure 5:**
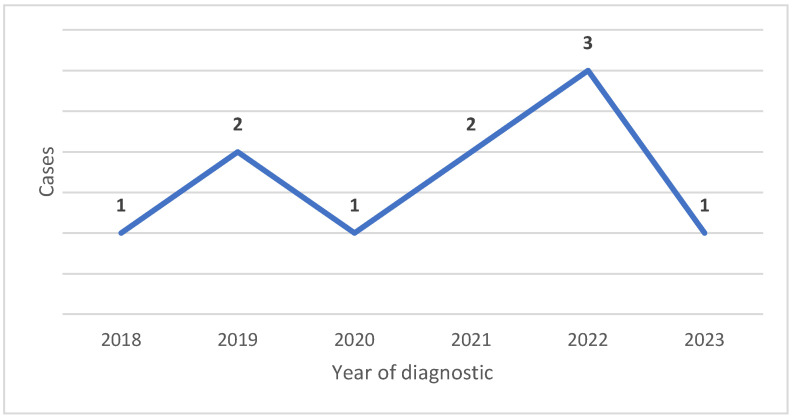
Distribution of Spitz tumors diagnosed between 2018 and 2024.

Figure 5 shows the case distribution through the study periods.

Patients were of various ages, with the extremities of age being 11 and 40 years. Table 2 shows the patient demographics.

**Figure 6:**
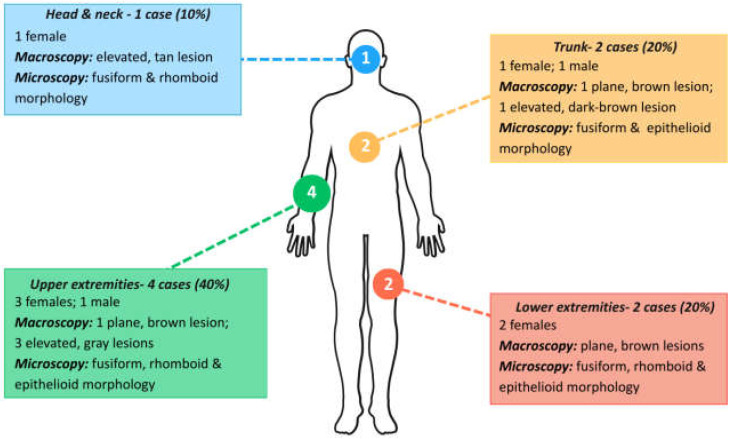
Distribution and characteristics of the Spitz lesions included in the study.

The area of the lesion and the macroscopical parameters of the lesions are presented in Table 3 and Figure 6.

**Figure 7:**
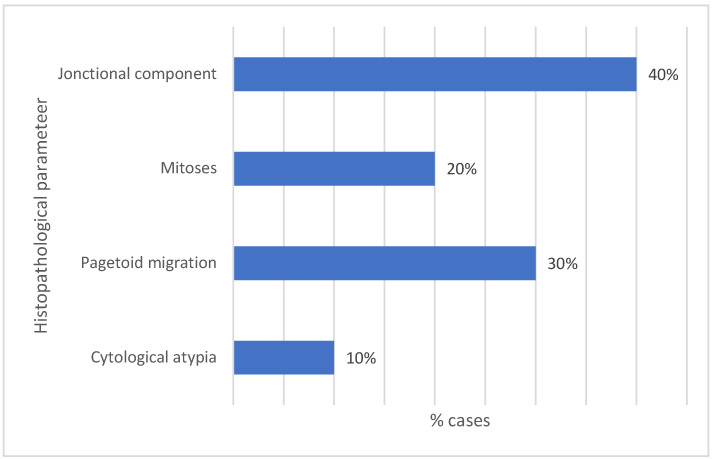
Percentual findings for the histopathological parameters of the Spitz lesions.

The histological characteristics of the Spitz lesions are presented in Table 4 and Figure 7.

The immunohistochemistry of each case is presented in Table 5.

## 4. Discussion

Spitz nevi are distinct, rare, melanocytic proliferations with mainly spindle or epithelioid morphologies that exhibit a heterogeneous clinical appearance, posing difficulties for both dermatologists and dermatopathologists [12,13]. Since being initially reported, their nomenclature has dramatically changed over the years. According to *WHO Classification of Skin Tumours* (5th ed) [1], the wide spectrum of Spitz neoplasms can be classified into three main categories: Spitz nevi, which include the Reed nevus, also called the pigmented spindle nevus and the typical Spitz nevus; the Spitz melanocytoma, which was previously called the atypical Spitz tumor; and the Spitz melanoma [5,14]. The aim of our study was to highlight the clinicopathological characteristics of 10 Spitz tumors diagnosed over a 6-year time frame at a university hospital in Romania.

The exact pathogenesis of Spitz tumors remains unclear; however, recent studies have identified a series of gene fusions and mutations that are responsible for their development. Although the Spitz nevus is a benign lesion, its peculiar characteristic lies in its close histopathological resemblance to melanoma rather than to a conventional nevus [6]. The genomic landscape of Spitz neoplasms includes fusions in genes such as ALK, ROS1, MET, RET, or BRAF and mutations in HRAS genes, which influence both the tumor’s biological behavior and morphological features [6,7,15,16]. Given the unique and complex morphology of Spitz lesions, contemporary approaches should focus on combining clinicopathological characteristics with ancillary studies such as immunohistochemistry and molecular testing to enhance diagnostic accuracy. Unfortunately, in our study, molecular testing was not available.

Spitz lesions represent benign melanocytic proliferations characterized by a distinctive cellular morphology that falls within the spindle–epithelioid spectrum. Figure 1 presents the main three cellular subtypes identified in our cases: epithelioid (Figure 1A); spindle-shaped (Figure 1A); and rhomboid (Figure 1C). Differentiating between Spitz nevi and melanoma can sometimes pose challenges to pathologists due to shared histological features between the two lesions. However, several clinicopathological criteria are used to guide diagnosis. Figure 2 illustrates the main histological characteristics of a classic Spitz nevus: Kamino bodies (Figure 2A); junctional nests (Figure 2B); epidermal hyperplasia (Figure 2C); pagetoid spread (Figure 2C); and maturation (Figure 2D).

Based on the distribution of melanocytes at the dermo-epidermal junction and/or within the dermis, Spitz naevi are classified as junctional, intradermal, or compound (involving both junctional and dermal components). Most Spitz naevi are either compound or intradermal. Figure 3 illustrates the most common type: compound Spitz nevus.

The histopathological assessment of Spitz naevi is primarily based on Hematoxylin and Eosin-stained slides; however, ancillary immunohistochemical markers are often employed to support the diagnosis in challenging cases. Figure 4 -left- shows a compound Spitz nevus in routine Hematoxylin and Eosin staining. Figure 4 -middle- and -right- displays immunohistochemical reactions used for assessing both the melanocytic lineage (SOX-10 nuclear staining) and proliferative activity (Ki-67 nuclear marker).

Microscopic evaluation of Hematoxylin and Eosin slides remains the gold standard for melanocytic neoplasm diagnosis. However, morphology alone might not be sufficient for the accurate assessment of challenging lesions, including Spitz tumors [6,7,15]. Therefore, immunohistochemistry can be used as an ancillary test for better nosological characterization and further establishment of the benign or malignant nature of Spitz lesions. Like other melanocytic tumors, these cells express positivity for S100, SOX10, Melan-A (MART1), tyrosinase, and MITF [5]. HMB45 has a lower expression in Spitz nevi, and usually the maximum Ki-67 index is 5% [8,15]. In some cases, the expression of p16 in Spitz nevi can be helpful in distinguishing between benign proliferations and Spitz melanoma; however, the loss of p16 may be seen in all three types of Spitz melanocytic proliferations [16,17]. Herein, in 9 out of 10 cases, melanocytic markers were assessed (Table 5). The main marker used was S100, which showed diffuse expression in eight cases of Spitz nevi. SOX-10 and Melan-A were performed in cases with indeterminate histopathological features to further confirm the melanocytic differentiation. In all the cases presented, the Ki-67 proliferation index was below 5%, with most tumors exhibiting a Ki-67 index between 1% and 2%. Additionally, Preferentially Expressed Antigen in Melanoma (PRAME) serves as both a diagnostic and prognostic marker, primarily used in the assessment of melanoma [18]. However, its expression in Spitzoid lesions is variable. Consequently, PRAME immunoreactivity is significantly lower in Spitz nevi and atypical Spitz tumors compared to their malignant counterparts [19]. Therefore, the interpretation of PRAME expression should always be correlated with the clinical context and histopathological features. Given the heterogeneous expression of HMB-45, p16, and PRAME in Spitzoid lesions, the diagnostic assessment of Spitz nevi in this study was primarily based on architectural and cytological criteria, supplemented by a limited immunohistochemical panel of melanocytic markers (including S100, SOX-10, and Melan-A/MART-1) available in a small-scale pathology laboratory.

As Spitz melanoma is classified as a non-CSD melanoma and most frequent and exclusive mutations are represented by BRAF and NRAS mutations in melanoma, the molecular profiling becomes even more important in terms of diagnosis and prognosis [20].

Spitz tumors which are associated with BRAF mutations are associated with worse outcomes [21]. Atypical melanocytic tumors with Spitzoid features can be classified by morphology, immunohistochemistry and FISH, but the mutational status of BRAF and NRAS brings a clearer view to this type of tumors. As Moysset et al. [22] described in its study, out of 71 atypical melanocytic tumors with Spitzoid features, after morphology, IHC and FISH investigations, 47 were classified as intermediate tumors with Spitzoid features and 24 where Spitzoid melanoma. The expanded molecular profiles have redefined the initial classification of the 71 atypical melanocytic tumors in ATS, BRAF-mutated nevus/melanocytoma (BAMS), Spitz melanoma and melanoma with Spitzoid features [22]. Although IHC, including MelanA, Ki67, HMB45 and p16 are good markers for ATS, if p16 expression is lost in ATS, CDKN2A mutation should be investigated. Prognostic value for the patient is added by always establishing the BRAF and NRAS profile for these lesions [2].

The p16 expression may by partial loss in cases which are histopathological difficult to classify between melanoma, Spitz nevus or AST [23]. Retained p16 expression with a negative result in the BRAF V600E mutations have lower levels of FISH chromosomal abnormalities related to melanoma in comparison to the expression loss of p16. BRAF V600E mutations in cases with retained p16 expression Spitz tumors can serve as an explanation of Spitzoid features in young patients; however, they exclude Spitz tumors [24,25].

Our study analyzed the macroscopic features and histological characteristics of nine Spitz nevi and one atypical Spitz tumor over a 6-year period (Figure 5). Consistent with findings from other studies, a significant decrease in skin neoplasms was observed in our research during the COVID-19 pandemic [26]. Based on all Spitz melanocytic lesions diagnosed in the pathology department, it can be seen from Table 2 that most of the patients were under 20 years old, with the oldest patient being 40 years old. In our study, the mean age at diagnosis was 20.2 years, which is in line with data in the current literature [1,16]. Spitz lesions usually appear in young people but may appear at any age. Reports suggest that Spitz nevi are slightly more common in female individuals [6,27,28]. Similarly, our study demonstrated a predominance of female gender among those with Spitz nevi.

In our study, most of the lesions were localized on the upper and lower limbs (Table 3 and Figure 6). This may be due to an increased prevalence in exposed regions, especially in children and young adults. All the flat lesions were brown and had small dimensions and well-defined margins, which are macroscopic characteristics of benign nevi. The elevated and prominent lesions were lighter in color, which may reflect a reduced density of melanocytic pigment or a deeper proliferation of melanocytic cells.

Most of the lesions were round, symmetrical, and well-defined, suggesting a benign appearance. Other forms, such as papillomatous or irregularly shaped lesions, were less frequent and indicated adjacent supplementary assessment. The brown lesions did not present any mitotic activity or pagetoid migration, suggesting a stable histological pattern. This observation suggests that the color and shape of lesions may serve as guiding parameters during the pre-surgical period.

Spitz nevi are histologically characterized by a proliferation of spindle-shaped or epithelioid melanocytes, which may be in the dermo-epidermal junction, within the dermis, or in both compartments [29]. Common histopathological features of Spitz nevi include vertically oriented nests, clefting around the junctional nests, the presence of Kamino bodies, and pagetoid spread, while mitotic activity, if present, is confined to the superficial dermis [1,30,31,32]. Most cases included in this research exhibited a mixed cellular morphology, comprising fusiform, rhomboid, or epithelioid components, and were classified as purely intradermal lesions (Table 4 and Figure 7). Cytological atypia was seen in 1/10 cases, mitoses were seen in 2/10 cases, and pagetoid migration was seen in 3/10 cases. Clinicopathological studies have demonstrated that although small lesions may exhibit significant histological atypia, their clinical risk remains low, whereas larger tumors are associated with unfavorable outcomes [9,15,32,33]. Consistently, in our study, we found that the presence of pagetoid spread and lesions’ dimensions are independent markers for atypical behavior, as both features were associated with a higher degree of cytological atypia in Spitz nevi.

Using the Pearson correlation coefficient, we can see a moderate positive correlation between pagetoid migration and cytologic atypia. The most significant correlation is between the tumor area and cytological atypia, where the Pearson coefficient is 0.733 with *p* = 0.0159 and 95% confidence intervals of [0.191, 0.932]. Thus, it can be stated that larger lesions are more likely to show cytological atypia, a potential marker for atypical behavior. Taking into consideration the small number of cases included in this study, the correlation findings should be interpreted with caution.

In terms of the anatomic distribution of the lesions, the topographic area varied. Most of the cases were located on the upper extremity (n = 4; 40%), followed by the lower extremity (n = 2; 20%) and the trunk (n = 2; 20%). In a study conducted by Uzuncakmak T., it was reported that the anatomical distribution of Spitz nevi varied with age, with a higher prevalence on the upper extremities in pediatric patients, while lower extremities were a more common site in adults [8]. Regarding gross examination, lesions exhibited varying clinical appearance. Half of the lesions were elevated from the skin, whereas the other half were plane lesions. Most Spitz nevi were less than 1.5 cm in diameter and varied greatly in color, from tan to dark brown. Likewise, in a study by Herzum et al. conducted on 255 pediatric patients, small, pigmented, flat lesions were found to be associated with benign morphological features [29].

Many lesions were excised within the safety excision margin. The atypical Spitz tumor had a microscopical excision margin of only 1 mm, emphasizing that its uncertain biological behavior necessitates increased attention, even though, macroscopically, the lesion was completely excised. The microscopic assessment of atypic lesions when molecular testing is not available remains a critical parameter for oncological safety. Our data suggests that strong collaboration between the surgeon and the pathologist is essential in achieving the best outcome for each patient. The Spitz nevus was the predominant histopathological diagnosis at our hospital, with a low incidence recorded for the atypical Spitz tumor. This distribution confirms the fact that most of the Spitz lesions are found in children and young adults, but there is a necessity for close follow-up for AST. The presented distribution offers insights into the practical classification of Spitz lesions in routine pathology, particularly in resource-limited pathology departments.

Limitations of the study: Our study has several limitations. Even considering that Spitz lesions are rare, the number of cases included in this study was small, since it involved just one county hospital in Romania. No molecular tests were performed, because our department does not have its own molecular testing unit. Another limitation is that only one center was included in the study. Taking into consideration the small number of cases included in the study, the statistical correlation findings should be interpreted with caution.

## 5. Conclusions

Our study represents a significant contribution to daily practice regarding Spitz tumors, with an emphasis on morphological and immunohistochemical parameters in the absence of molecular testing. A systematic approach based on macroscopic, microscopic, and immunohistochemical methods can play a significant role. This study is descriptive in nature, and clinicopathological assessment is valuable, but there is an increasing need for it to be complemented with genomic testing. The majority of the diagnosed Spitz tumors were characterized by Spitz nevi, confirming the benign predominance in our study population. The patients were predominantly young, emphasizing the juvenile character of Spitz tumors encountered in daily practice. Female gender was frequently observed. Histopathologically, most of the cells were fusiform or epithelioid, and pagetoid migration was seen in a few cases, not necessarily associated with aggressive behavior in the tumor. All the included lesions had a low Ki 67 index, and all the cases had free excision margins without the need for other surgical interventions. This study lays a foundation for future research, which could include molecular testing.

## Figures and Tables

**Table 1:** Comparative histopathological features of Spitz nevus, atypical Spitz tumor (AST), and Spitzoid melanoma [1,5,11].

Histopathological Feature	Spitz Nevus	Atypical Spitz Tumor (AST)	Spitzoid Melanoma
Size	<6 mm	6–10 mm	>1 cm
Symmetry	Symmetrical	May be symmetrical or asymmetrical	Asymmetrical
Circumscription	Well circumscribed	Well or poorly circumscribed	Poorly circumscribed
Ulceration	Rare	More common than SN	Present
Epithelioid/Spindle Cell Morphology	Can be epithelioid, spindle cell, or both; nests in “banana bunch” pattern	Epithelioid, spindle cell, or both; AST with MAP3K8: pleomorphism, increased N:C ratio	Epithelioid/spindle/both; irregular nests; ALK/NTRK1 fusions; MAP3K8: increased epithelioid morphology
Pagetoid Spread	Uncommon, typically focal if present	Peripheral, may involve upper epidermis	Present, may be extensive
Kamino Bodies	Present at periphery of melanocyte nests	Infrequent, smaller	Rare
Melanocyte Maturation	Present	May be present	Absent
Mitotic Activity	Low, 0–2/mm^2^	Moderate, 2–6/mm^2^	High, >6/mm^2^
Lymphocytic Inflammatory Infiltrate	Commonly present	May be present	May be present
Multinucleation	May be present; often in epithelioid SN	May be present (MAP3K8-associated)	Rare
Epidermal Hyperplasia	Present	Rare	Rare

**Table 2:** Year of diagnosis, age, and gender of patients diagnosed with Spitz tumors.

Patient No.	Year of Diagnosis	Age (Years)	Gender
1	2018	26	Male
2	2019	19	Female
3	2019	17	Female
4	2020	19	Female
5	2021	11	Female
6	2021	11	Male
7	2022	40	Female
8	2022	23	Female
9	2022	23	Female
10	2023	13	Male

**Table 3:** Body location and macroscopic parameters of Spitz lesions.

Patient	Area	Macroscopic Appearance	Macroscopic Dimension (mm)	Macroscopic Volume or Circumference *	Macroscopic Shape	Macroscopic Color
1	Right arm	Plane lesion	4 × 4	14.17 mm	Round	Brown
2	Left calf	Plane lesion	3 × 2	8.68 mm	Round	Brown
3	Nasal pyramid	Elevated from skin surface	5 × 5 × 2	7.97 mm^3^	Nodular	Whitish-gray
4	Left arm	Elevated from skin surface	5 × 5 × 2	7.97 mm^3^	Round	White-gray
5	Upper limb	Elevated from skin surface	13 × 12 × 4	28.18 mm^3^	Round	Whitish
6	Right forearm	Elevated from skin surface	5 × 7 × 2	9.44 mm^3^	Papillomatous	Whitish
7	Unspecified	Plane lesion	3 × 3	10.63 mm	Round	Brown
8	Dorsal portion	Plane lesion	7 × 6	22.97 mm	Round	Brown
9	Right buttock	Plane lesion	5 × 4	15.85 mm	Round	Brown
10	Posterior thorax	Elevated from skin surface	3 × 3 × 0.5	7.51 mm^3^	Unregulated	Dark brown

* When three dimensions were available, the volume was calculated; when two dimensions were available, the circumference was calculated.

**Table 4:** Main histopathological characteristics of the Spitz lesions.

Patient	Cells Shape	Cytological Atypia	Pagetoid Migration	Mitoses	Junctional Component
1	Fusiform	No	No	No	Yes
2	Fusiform, rhomboid, epithelioid	No	Yes	Yes—4	Yes
3	Fusiform, rhomboid	No	No	No	No
4	Fusiform, rhomboid	No	No	No	Yes
5	Fusiform, rhomboid, epithelioid	Yes	No	Yes—6	No
6	Fusiform, rhomboid, epithelioid	No	No	No	Yes
7	Fusiform, epithelioid	No	Yes	No	No
8	Fusiform, epithelioid	No	Yes	No	No
9	Fusiform, epithelioid	No	No	No	No
10	Fusiform, epithelioid	No	No	No	No

**Table 5:** Immunohistochemistry profile of each of the Spitz lesions.

Patient	Immunohistochemistry	S100	SOX	Melan A	Ki 67
1	Yes	Positive in nevus cells	NP *	Positive in nevus cells	1–2% in tumoral cells
2	Yes	Positive in nevus cells	NP *	Positive in nevus cells	Under 5% in tumoral cells
3	Yes	Positive in nevus cells	NP *	NP *	Under 5% in tumoral cells
4	Yes	Positive in nevus cells	NP *	NP *	1–2% in tumoral cells
5	No	NP *	NP *	NP *	NP *
6	Yes	Positive in nevus cells	Positive in nevus cells	Positive in nevus cells	1–2% in tumoral cells
7	Yes	Positive in nevus cells	Positive in nevus cells	Positive in nevus cells	Under 5% in tumoral cells
8	Yes	NP *	Positive in nevus cells	NP *	1–2% in tumoral cells
9	Yes	Positive in nevus cells	NP *	NP *	1–2% in tumoral cells
10	Yes	NP *	NP *	NP *	1–2% in tumoral cells

* NP, not performed.

## Data Availability

Data available on request due to privacy reasons.

## Abbreviations

The following abbreviations are used in this manuscript:

ALK	Anaplastic Lymphoma Kinase
BRAF	B-Raf Proto-Oncogene
Non-CSD	non- cumulative solar damage
HMB45	Human Melanoma Black 45
Ki 67	Cell Proliferation Marker Ki-67
MAPK	Mitogen-Activated Protein Kinase
MART1	Melanoma Antigen Recognized by T-cells 1
MITF	Microphthalmia-Associated Transcription Factor
Melan-A	Melanoma Antigen A
NTRK1	Neurotrophic Tyrosine Receptor Kinase 1
ROS1	ROS Proto-Oncogene 1
SOX-10	SRY-Box Transcription Factor 10
SOX10	SRY-Box Transcription Factor 10
TERT	Telomerase Reverse Transcriptase
USA	United States of America
p16	Cyclin-Dependent Kinase Inhibitor 2A
PRAME	Preferentially Expressed Antigen in Melanoma
